# Case Report of an open Neck Procedure Complication Associated with Laryngeal Mask Airway Use

**Published:** 2018-03

**Authors:** Thavakumar Subramaniam, Paula Casserly

**Affiliations:** 1 *Department of Otolaryngology-Head and Neck Surgery, St James Hospital, Dublin. Ireland.*

**Keywords:** Complication, Laryngeal mask airway, Pharyngeal distortion

## Abstract

**Introduction:**

The laryngeal mask airway (LMA) is a safe method to establish airway control during general anaesthesia. In otolaryngology surgery, the use of a LMA is well established in ear surgery; however, the use of a LMA during open neck procedures remains controversial. We report a case in which the pharyngeal distortion by the LMA cuff resulted in an iatrogenic complication.

**Case Report:**

A 38-year-old female with a background of multiple myeloma was referred to the otolaryngology team for an open cervical lymph node biopsy. The patient was in remission after a 2 year post chemotherapy treatment, but now presented with a 4-week history of persistent nodal enlargement. During the elective procedure, pharyngeal distortion from the laryngeal airway mask used for airway management resulted in an iatrogenic pharyngeal injury. This case is reported to highlight the importance of communication between the surgeon and anesthetist about the mode of airway management in open neck surgery.

**Conclusion:**

Communication between the otolaryngologist and anesthetist is pertinent when selecting the method of airway management in open neck procedures. A LMA should be used with caution during open neck procedures, with the surgeon recognizing the potential for pharyngeal distortion.

## Introduction

The laryngeal mask airway (LMA) is a safe method to establish airway control during general anaesthesia. In otolaryngology surgery, the use of a LMA is well established in ear surgery; however, the use of a LMA during open neck procedures remains controversial. We report a case in which the pharyngeal distortion by the LMA cuff resulted in an iatrogenic complication.

## Case Report

A 38-year-old female with a four-week history of left level II cervical lymph node enlargement was referred to the Ear Nose and Throat (ENT) team for open neck biopsy. She had a history of multiple myeloma treated with chemotherapy and autologous bone marrow transplant two years prior to referral. The referring hematology team were concerned with a secondary malignancy. 

Complete head and neck examination revealed a palpable level 2 lymph node but was otherwise normal. A computer tomography of the neck was performed and showed an isolated two centimeter cervical lymph node correlating to clinical examination with no other significant findings. An ultrasound guided fine needle aspiration was performed on the node. Unfortunately, the cytology was reported as non-diagnostic and the patient was scheduled for open biopsy.

Pre operative platelet infusion was required as the patient had chronic thrombocytopenia. The anaesthetic team commenced general anaesthetic induction after the post infusion platelet count was confirmed to be 45 x 10^3^/mL. Although higher platelet levels would have been preferred, this was not possible even with repeated platelet infusions. The ENT team anticipated a possible concern with hemostasis, but opted to proceed with the procedure due to the potential malignant underlying pathology. The expected duration of the procedure was confirmed to be thirty minutes by the operating ENT team. 

A two-centimeter incision was made followed by a deeper dissection. The anterior border of the sternocleidomastoid muscle (SCM) was retracted and a compressible mass was palpable medial to the muscle. Fascia over the palpable mass was skeletonized off to reveal a thin-walled air filled mass that was initially thought to be a possible laryngocoele. No other abnormal lymph nodes were palpable. At this stage, the operating surgeon realized that the “air filled mass” was actually the LMA and the pharyngeal wall had been dissected, resulting in a one centimeter iatrogenic pharyngeal tear. Prior to continuing with the procedure, the surgeon requested that the LMA be replaced with an ETT. The one centimeter pharyngeal tear was repaired and exploration showed no further damage. The patient was placed on total parenteral nutrition (TPN) and on intravenous broad spectrum antibiotics. A gastro-graffin swallow test done on day 10 post-operation showed no leakage and the patient was commenced on an oral diet without any issues. Post-operative imaging revealed that the original enlarged lymph node now measured less than 1cm. Intra operative events were discussed with the patient in full disclosure. The patient was subsequently referred back to the hematology team who opted to follow up the patient with serial imaging. 

## Discussion

The LMA was invented by British anesthesiologist Dr. Archie Brain and has revolutionized anaesthetic airway management since its introduction into clinical practice in the 1980s (1). The LMA was designed so that the cuff be placed extending from the base of the tongue to the upper oesophageal sphincter, bounded laterally by the piriform fossae, and protecting the glottic and supraglottic structures (1,2). Although rare, inappropriate cuff size selection or mal positioning can result in complications such as sore throat, laryngospasm, aspiration, and dislodgment (3,4). There have been complications reported due to local pressure effect from the LMA cuff such as recurrent laryngeal nerve palsy, lingual nerve palsy, hypoglossal nerve palsy, submandibular gland inflammation, and parapharyngeal and retropharyngeal abscess (3,5-11). Several factors lead to the undesired complication in our case. Despite communication between the ENT and anaesthetic team prior the procedure, airway management was not discussed, as the use of ETT was standard in all open neck procedures in our center. However, the LMA was used as the duration of the procedure was expected to be less than thirty minutes and the further influence of the patient’s low platelet count. In our case, operative limitations were the use of a relatively small incision, hemostasis concerns with the patient’s thrombocytopenia, and the cachectic status of the patient. Minimal muscle dissection and instrumentation lead resulted in the pharyngeal perforation.

Samuels et al reported a similar case to ours, where pharyngeal distortion or bulge from the cuff occurred because of the LMA (12). The author reports a case whereby the pharyngeal bulge from the LMA cuff was mistaken for cervical lymphadenopathy during an open biopsy of a clavicle mass. This resulted in an unnecessary fine needle aspiration being performed. 

A review article by Jefferson et al highlights the safety and efficacy of LMA use during various ENT procedures (2). The author does recognize however the dangers of LMA use during open neck procedures especially when there is no shared consensus on airway management between the ENT and anaesthetic team. Similarly to our case, Jefferson et al highlights that the mistake is only evident once the LMA cuff is encountered in the open neck (2). 

## Conclusion

The importance of communication between an otolaryngologist and anesthesiologist is best described by Jefferson et al “In no other branch of surgery is the relationship between anaesthetist and surgeon closer than in otorhinolaryngology” (2). 

Although several factors may have contributed to the occurrence of this complication, detailed communication on all aspects of peri-operative care is paramount and key to avoiding such incidents. The use of a LMA in open neck procedures remains controversial. 

We present this case to highlight an iatrogenic complication to reiterate the potential danger of using a LMA in an open neck procedure. 
